# Methodological Considerations in Development of UV Imaging for Characterization of Intra-Tumoral Injectables Using cAMP as a Model Substance

**DOI:** 10.3390/ijms23073599

**Published:** 2022-03-25

**Authors:** Frederik Bock, Johan Peter Bøtker, Susan Weng Larsen, Xujin Lu, Jesper Østergaard

**Affiliations:** 1 Department of Pharmacy, Faculty of Health and Medical Sciences, University of Copenhagen, Universitetsparken 2, DK-2100 Copenhagen, Denmark; frederik.bock@sund.ku.dk (F.B.); johan.botker@sund.ku.dk (J.P.B.); susan.larsen@sund.ku.dk (S.W.L.); 2 Bristol Myers Squibb Company, Drug Product Development, 1 Squibb Drive, New Brunswick, NJ 08901, USA; xujin.lu@bms.com

**Keywords:** cAMP, injectable, in vitro release testing, intra-tumoral administration, local administration, UV imaging

## Abstract

A UV imaging release-testing setup comprising an agarose gel as a model for tumorous tissue was developed. The setup was optimized with respect to agarose concentration (0.5% (*w*/*v*)), injection procedure, and temperature control. A repeatable injection protocol was established allowing injection into cavities with well-defined geometries. The effective resolution of the SDi2 UV imaging system is 30–80 µm. The linear range of the imaging system is less than that of typical spectrophotometers. Consequently, non-linear cAMP calibration curves were applied for quantification at 280 nm. The degree of deviation from Beer’s law was affected by the background absorbance of the gel matrix. MATLAB scripts provided hitherto missing flexibility with respect to definition and utilization of quantification zones, contour lines facilitating visualization, and automated, continuous data analysis. Various release patterns were observed for an aqueous solution and in situ forming Pluronic F127 hydrogel and PLGA implants containing cAMP as a model for STING ligands. The UV imaging and MATLAB data analysis setup constituted a significant technical development in terms of visualizing behavior for injectable formulations intended for intra-tumoral delivery, and, thereby, a step toward establishment of a bio-predictive in vitro release-testing method.

## 1. Introduction

Cyclic dinucleotides (CDNs) are stimulator of interferon genes (STING) agonists that lead to increased inflammatory response against pathogens and tumors [1]. Recently, naturally occurring and synthetic CDNs have attracted attention due to the implication of STING in cancer and inflammation in various autoimmune and metabolic diseases. Clinical trials with CDNs are ongoing; however, the anti-tumor efficacy observed in mice has not been replicated in humans [2]. Absence or saturation of the transporter SLC19A in human tumor tissue might explain this, as the transporter is responsible for CDN uptake in humans [2]. Cytosolic delivery of CDNs is necessary, as STING is located in the cytosol [1], and improved cytosolic delivery of CDNs using drug delivery systems such as hydrogels or nanoparticles might promote efficacy in humans [1,2]. CDNs have a molecular weight of ~700 g/mol, are subject to extracellular enzymatic cleavage, and are highly polar (double negative charge at physiological pH), which makes cytosolic delivery challenging due to poor membrane permeability [1]. A high, localized concentration of the CDN 2′,3′-cGAMP in a tumor is necessary to achieve anti-tumor effect [3]. This makes intra-tumoral (IT) administration of controlled release formulations interesting, as they can be designed to provide high local concentration inside the tumor for a prolonged period while reducing dosing frequency [3]. An in situ forming controlled release formulation of 2′,3′-cGAMP showed anti-tumor effect in mice after a single IT injection [3,4].

Drug release from in situ forming formulations into a hydrogel phantom mimicking the injection environment may be more suitable for predicting in vivo behavior as compared to release into a bulk solution [5]. Agarose gels have been applied as tissue-mimicking phantoms for tumors [6,7] and subcutaneous tissue, e.g., in [8,9,10,11,12]. Agarose phantoms have a pore size of 600–1200 nm and resemble subcutaneous tumors [6]. These tissue-mimicking phantoms are compatible with imaging techniques such as ultrasound, photoacoustic, fluorescence, and magnetic resonance imaging (MRI) [13,14]. Imaging techniques such as MRI, near-infrared imaging, and UV imaging are increasingly used in formulation design and development to visualize dissolution and release behavior [15,16,17]. In UV imaging, temporally resolved absorbance maps are generated utilizing the absorbance properties of an analyte [15]. Second-generation UV imaging devices can record simultaneously at wavelengths in the UV and visible ranges [15,18]. Hydrogel matrixes have been used together with UV imaging to determine drug diffusion [19] and drug release from different vehicles into hydrogels [11,20,21]. UV imaging has been used to characterize dissolution behavior, including determination of intrinsic dissolution rates for active pharmaceutical ingredients [22,23]. UV imaging has also found use in characterizing the performance of injectable formulations [15,18]. With the growing interest in parenteral sustained-release formulations, in vitro methods, which accurately model the conditions at the injection site, are needed. As of today, there are no compendial in vitro release methods for conventional- or controlled-release parenteral formulations [24,25,26].

The aim of this study was to develop a UV-imaging-based in vitro release method suitable for screening nucleotide formulations with an in-house-developed release cell and data processing methodology. UV imaging, for which the SDi2 system was used, was characterized with respect to performance and suitability related to local (intra-tumoral) administration. The release of cAMP from selected formulations was investigated in the process of developing the UV-imaging-based release-testing platform. cAMP is a second messenger, and changes in the intracellular concentration regulate several processes in the body [27]. cAMP was used as a model compound for CDNs, as it structurally resembles 2′,3′-cGAMP and possesses relatively similar physical-chemical properties: log *P* = −2.96, p*K*_a1_ = 1.39, and p*K*_a2_ = 3.81 for cAMP [28,29], and log *P* = −3.78 ± 3.16 [30], p*K*_a1_ = 0.89 ± 0.70, and p*K*_a2_ = 3.63 ± 0.10 [31] for 2′,3′-cGAMP (predicted values from AlogPS 2.1 and Advanced Chemistry Development (ACD/Labs) Software).

## 2. Results and Discussion

### 2.1. Initial Experiments—Development of In Vitro Release Setup for Static Conditions

Release, distribution, and diffusion of drug substances in extracellular matrix (ECM) mimics have mainly been studied in so-called small-scale cells [11,15,16]. However, accommodation of the administration step related to characterization of injectables by UV imaging, as well as by other techniques, remains challenging.

#### 2.1.1. Release Cell

**Figure 1:**
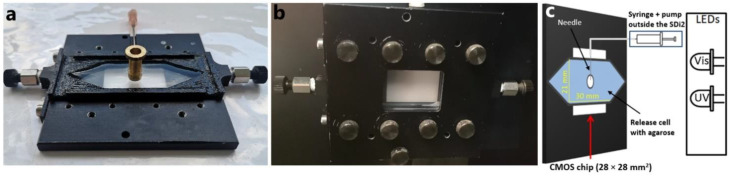
Experimental setup. (**a**) 3D-printed release cell filled with agarose gel and metal cylinder creating the cavity. (**b**) Release cell inside metal casing mounted in the SDi2. (**c**) Schematic representation of the UV imaging in vitro setup. The needle inserted into the cavity inside the agarose gel was connected to a syringe pump.

A key element in developing a prototype UV imaging release setup allowing the injection of formulations into an agarose gel under static conditions is the release cell. Using an in-house-built flow cell [32] as the starting point, a 3D-printed polylactic acid (PLA) cell and metal casing was designed for mounting in the SDi2 system (Figure 1). Commercially available 75 × 26 × 1.0 mm^3^ (*L* × *W* × *D*) quartz plates were sandwiched between the PLA walls and the aluminum casing to form the walls of the cell. The cell was equipped with an inlet and an outlet facilitating use as a flow-through cell. Furthermore, access for injection of the formulation was provided (Figure 1).

The 3D printing of PLA cells is ideal for prototyping (the cells are suited for repeated use), allowing flexible design and design changes. Leakage was the main challenge encountered using the cartridges. A seal between the quartz and the PLA walls was achieved using high-vacuum silicone grease. Future developments might include the introduction of a dedicated injection port, further preventing evaporation and/or leakage of medium.

#### 2.1.2. Matrix and Injection

Agarose gels have been used to mimic subcutaneous tissue [8,9,10,11,12] as well as tumor tissue (tissue-mimicking phantoms) [6,7] and were selected for development of the static UV imaging release setup. An agarose concentration of 0.5% (*w*/*v*) was found optimal (mainly based on the optical properties), resulting in adequate gel strength and providing reference images with lower background absorbance as compared to a 1% (*w*/*v*) agarose gel. The agarose gel was sufficiently transparent at the SDi2 relevant wavelengths (255, 280, 300, 320, and 520 nm). Initial experiments with injection of formulations into the release cell filled with the agarose gel revealed that the formulation occasionally spread along the needle and out of the injection entry, presumably due to rigid walls of the cell leading to pressure buildup. It was found that injection into a pre-made cavity alleviated the issue and improved the repeatability of the injection step. The preferred injection protocol (used in the release experiments detailed in Section 2.3 and Section 3.3) utilized a needle, injecting the formulation into the cavity filled with buffer. This protocol allowed repeatable injection of formulations with different viscosities, varying the needle size (diameter) while avoiding entrapment of air in the cell. Furthermore, the setup provided the opportunity to confine the formulations to the desired geometry. The injections were performed using a programmable syringe pump, except for the in situ forming poly(d,l-lactide-*co*-glycolide) acid (PLGA; 50:50) formulation, which was injected manually.

#### 2.1.3. Temperature Control

The majority of UV imaging studies reported so far were performed at ambient temperature. The SDi2 instrument is not intended for static experiments, i.e., the temperature control and heating pads are designed for flow-through experiments. Improved temperature control during release experiments was achieved by moving the SDi2 instrument into a temperature-controlled room (22 °C), allowing a temperature within the agarose matrix of 37 ± 1 °C to be maintained throughout the experiments.

### 2.2. Instrument Performance and Image Analysis

As part of the development of the prototype setup, the performance of the SDi2 UV imaging system was characterized in relation to resolution and dynamic range as well as image analysis capabilities.

#### 2.2.1. Resolution

The spatial resolution of the SDi2 was estimated to 30 µm in the x and y directions at optimal imaging conditions, i.e., with the grid directly placed at the CMOS sensor (Supplementary Materials Figure S1a). The resolution of UV imaging instruments is dependent on the positioning of the image objects relative to the CMOS sensor surface and the light source [33]. At the least optimal imaging condition (grid placed 12 mm in front of the CMOS sensor with cell holding 0.5% (*w*/*v*) agarose gel in between), resolution was estimated to 80 µm in the x and y directions (Supplementary Materials Figure S1b). The effective resolution during release experiments using the setup would be between the determined values. In line with our previous experiences with UV imaging instrumentation, the resolution is lower than the effective, nominal pixel size (13.75 × 13.75 µm^2^) of the CMOS detector. This is important to bear in mind when considering nano- and microparticle formulations.

#### 2.2.2. Measuring Range

**Figure 2:**
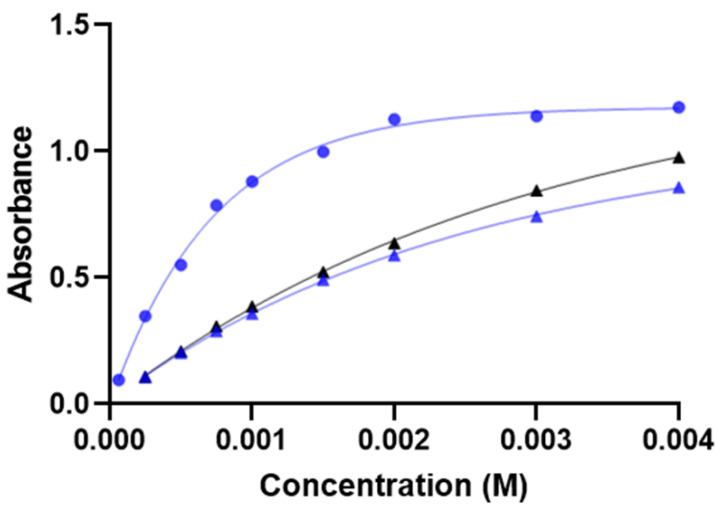
Calibration curves for cAMP in 0.5% (*w*/*v*) agarose gel (●) in the release cell (4 mm light path). Calibration curves for cAMP in 20% (*w*/*w*) Pluronic F127 hydrogel (▲) and 0.5% (*w*/*v*) agarose gel (▲) in the diffusion cell (1 mm light path). All measurements were performed at 280 nm and 37 °C using the SDi2. The lines represent the least-squares fit to Equation (1) (R^2^ = 0.99).

UV–Vis imaging is based on the spatially and temporally resolved measurement of light intensities, which are subsequently recalculated into absorbance. Thus, UV–Vis imaging is in principle similar to UV–Vis spectrophotometry [33,34], where linear relations between absorbance and concentration are commonly established under ideal conditions, i.e., Beer´s law. Figure 2 shows calibration curves for cAMP established in 0.5% (*w*/*v*) agarose gel and 20% (*w*/*w*) Pluronic F127 hydrogel at 280 nm using either the release cell or the so-called diffusion cells (Section 3.2). The cAMP concentration ranges explored expanded beyond the linear range in the release cell as well as in the diffusion cell. Non-linear calibration curves were obtained by fitting the data to Equation (1) (Section 3.4.2), which was used to convert absorbance to concentration of cAMP in the release experiments.

The measurement range of the SDi2 is narrow for cAMP at 280 nm, and the shape of the calibration curves depends on the sample matrix, e.g., agarose gel versus Pluronic F127 hydrogel. The UV imaging detection principle involves the conversion of UV light by a phosphorous layer to visible light detected by the CMOS [33]. The cause of the difference between the calibration curves can be discovered by inspection of Equation (2) (Section 3.4.2), used for calculating the absorbance from the pixel intensities. In 0.5% (*w*/*v*) agarose gel, *P*_0_ (pixel intensity for reference images) and *P*_s_ (pixel intensity for dark images) were typically 0.63 and 0.14 (MATLAB double format), respectively (4 mm light path). Reference images used for determination of *P*_0_ were obtained for the various matrixes. The absorptivity of the 0.5% (*w*/*v*) agarose gel was higher than that of the 20% (*w*/*w*) Pluronic F127 hydrogel, which led to a lower *P*_0_ for the agarose gel. In line with Equation (2), a more pronounced curvature was observed for the calibration curves in 0.5% (*w*/*v*) agarose gel as compared to that obtained in 20% (*w*/*w*) Pluronic F127 hydrogel (Figure 2). Consequently, the ECM mimic should have as low an absorptivity as possible (background absorbance) to have as wide a working range as possible for the calibration curves. Equation (2) is analogous to the equation used to account for stray light, which leads to deviation from Beer´s law in spectrophotometry. In principle, the linear range of the calibration curve can be extended by increasing the lamp power/light intensity. However, this study adhered to the settings recommended by the manufacturer. A potential additional contributing factor to the deviation from linearity is related to UV measurements performed at 280 nm on the slope of the cAMP spectrum rather than at a maximum (see Supplementary Materials Figure S2). Selection of 255 nm as the measuring wavelength would allow measurements near the absorbance maximum of cAMP. At 255 nm, however, the cAMP molar absorptivity as well as the background absorbance of the gel matrix were higher, which led to (more pronounced) deviation from Beer´s law. Hence, 280 nm was considered the better choice among the relevant wavelengths. For all the release experiments, ≤3% of the active image area accounted for pixel absorbance values ≥1 at any given time-point. The contribution from pixel absorbance values ≥1 accounted for ≤1% of the cAMP in the image area for the aqueous solution, Pluronic F127 hydrogel, and PLGA (50:50) implant and 4–12% for the PLGA (75:25) implant. cAMP concentrations measured in most of the imaging area were well below the plateau of the calibration curve. It is possible to use non-linear calibration curves; however, care should be exercised, as the sensitivity of the method decreases with higher concentrations and quantification errors may be introduced (as in the present case). Here, the importance of the background absorbance of the gel matrix was fully elucidated for the first time, and the significance of Equation (2) in predicting the shape of calibration curves was demonstrated. In addition to UV imaging at 280 nm, Vis imaging at 520 nm was performed. As cAMP does not absorb light at this wavelength, it was possible to visualize features of the formulation without interference of the drug, as discussed in Section 2.3.

#### 2.2.3. Image Analysis

**Figure 3:**
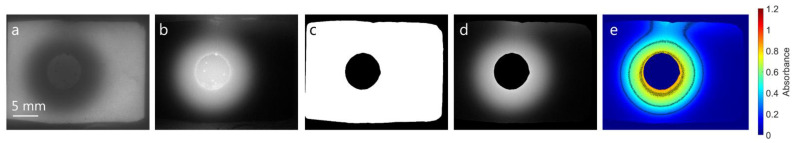
Example of image analysis work flow for an aqueous solution of cAMP (5 mg/mL) in 0.5% (*w*/*v*) agarose at 1 h after injection. (**a**) Gray-scale intensity image. (**b**) Absorbance image. (**c**) Auto-generated binary image used to remove cavity and edges (cavity area; the edges are automatically defined for each experiment (black = 0 and white = 1). (**d**) Absorbance image with cavity and edges removed. (**e**) Color-scaled absorbance image with contour lines.

Flexible and automated image analysis, beyond the capabilities of the commercially available software, was facilitated by the development of MATLAB scripts. The step-by-step process is outlined in the following, and the developed scripts can be found in Supplementary Materials (Figures S3 and S4). Intensity images (sample image shown in Figure 3a) were converted to absorbance images (Figure 3b) using Equation (2). An auto-generated binary image (Figure 3c) provided flexible analysis in regards to definition of quantification zones by multiplying the binary image with the absorbance images, thereby removing the cavity and edges (Figure 3d). The threshold values for the binary image can be adjusted to fit different experimental setups, providing flexibility in defining quantification zones (here, the objective was to quantify cAMP present in the gel matrix). The resulting absorbance images (sample image shown in Figure 3d) were converted to cAMP concentrations using the calibration curve constructed in the matrix used (here, 0.5% (*w*/*v*) agarose gel). The amount of cAMP for each pixel was calculated using the pixel dimensions and light path and subsequently summed for all pixels to determine the total amount of cAMP in the imaging area. The recorded images were processed in this manner (Figure 3a–d) using a conventional for-loop function in the MATLAB script. The automated image analysis facilitated analysis of all images; hence, it provided detailed insight of the release profiles. This is in contrast to the essentially manual or handheld approaches utilized to this point, in our lab and elsewhere, which effectively limited the exploitation of imaging data to include only a tiny fraction of the information recorded. A separate script was developed for the implementation of a color scale and contour lines on selected images. The color scale and contour lines visualized the transport pattern of cAMP upon release from the formulations and facilitated qualitative comparison between formulations (Figure 3e).

### 2.3. UV Imaging Release Experiments

**Figure 4:**
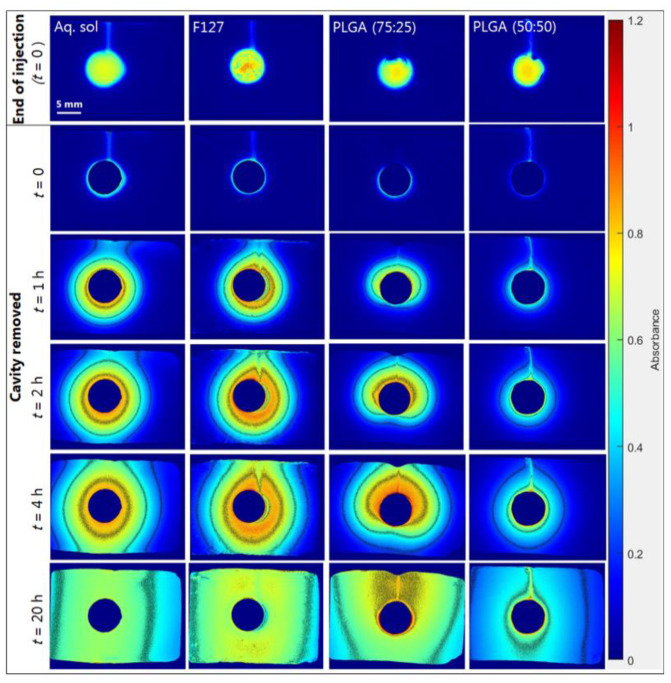
Representative absorbance images obtained at 280 showing the release of cAMP from an aqueous solution, an in situ forming Pluronic F127 hydrogel, a PLGA (75:25) implant, and a PLGA (50:50) implant into agarose gel at selected time-points and 37 °C. Contour lines represent absorbance values of 0.25, 0.5, 0.75, and 1. End of injection was the first image recorded, after instillation of the formulation was complete with the needle removed (defined as *t* = 0). Cavity removed referred to absorbance images, where the cavity area was excluded by multiplying with the auto-generated binary image.

The model substance cAMP was selected as a surrogate for STING ligands, which may be given by intra-tumoral (IT) injection. Four different formulations containing cAMP were prepared: an aqueous solution, an in situ forming 20% (*w*/*w*) Pluronic F127 hydrogel, an in situ forming 40% (*w*/*w*) PLGA (75:25) implant, and an in situ forming 33% (*w*/*w*) PLGA (50:50) implant (Section 3.1). Selected absorbance images from the release experiments are depicted in Figure 4. Gel matrixes are known to suppress density effects, i.e., the natural convection occurring in stagnant solutions [33]; thus, a symmetrical diffusion pattern surrounding the injection cavity was a priori expected. For the aqueous solution, a symmetrical transport or diffusion pattern of cAMP was observed for early time points, followed by development of an asymmetrical distribution pattern. The asymmetry was a result of the cell geometry, as the dimensions of the release cell are considerably larger in the horizontal direction than in the vertical direction (Figure 1c); as a result, cAMP is reflected at the horizontal boundaries (top and bottom walls), leading to the asymmetrical concentration distribution. The Pluronic F127 hydrogel exhibited an almost symmetrical transport pattern. The slightly asymmetric concentration distribution was most likely due to a fraction of the formulation ending up in the injection channel (needle track) upon injection. Upon injection of the in situ forming 40% (*w*/*w*) PLGA (75:25) formulation, a significantly different release pattern was found as compared to the other formulations. This may be a result of the in situ forming process involving NMP efflux and water influx from the injected PLGA (75:25)-NMP solution. The PLGA implant formation was imaged at 520 nm, where cAMP does not absorb light, and showed that the implant formation occurred from the bottom of the cavity and up for the PLGA (75:25) formulation (Supplementary Materials Figure S5). From the absorbance images, it was evident that the PLGA (50:50) implant exhibited a slower cAMP release than the other formulations (Figure 4). Furthermore, images at 520 nm revealed that the implant formation process was different for the two PLGA implants, as indicated by the optical densities during the in situ implant formation process (Supplementary Materials Figure S5).

**Figure 5:**
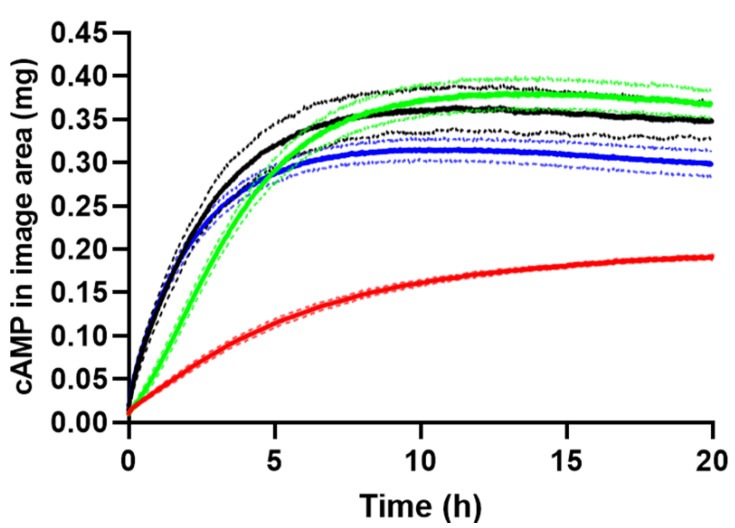
Amount of cAMP in the image area outside the cavity. Apparent release profiles for the aqueous solution (blue), the in situ forming Pluronic F127 hydrogel (black), the PLGA (75:25) implant (green), and the PLGA (50:50) implant (red) at 37 °C. Average of 3 replicates are in bold, and error bars (1 SD) are represented by the dotted lines.

The total amount of cAMP released to agarose gel in the image area was calculated using the established image analysis procedures and depicted in the apparent release profiles in Figure 5. The initial cAMP release from the PLGA (75:25) implant was significantly slower than release from the aqueous solution and the Pluronic F127 hydrogel. The amount of cAMP released from the PLGA (50:50) implant was significantly lower after 20 h compared to the other formulations. cAMP is negatively charged at physiological pH and has a log *P* of −2.96 [28]. The high polarity or hydrophilicity may explain the significantly slower release observed for PLGA (50:50) implant as compared to the PLGA (75:25) implant, as a lower lactic acid content results in a less hydrophobic copolymer [35], thereby retaining cAMP more effectively. The PLGA (50:50) implant has a higher viscosity than the PLGA (75:25) implant and may also result in a slower cAMP release [36]. Furthermore, faster depot formation was observed for the PLGA (50:50) implant compared to the PLGA (75:25) implant, as seen by the higher absorbance value at 520 nm (Supplementary Figure S5). The PLGA grades of 85:15, 75:25, and 50:50 (lactic acid:glycolic acid) are the most commonly used PLGA grades [37]. The PLGA 85:15 grade was excluded from this study because of the high polarity of cAMP, which therefore most likely renders this PLGA quality unsuitable for a sustained-release cAMP formulation, as the results outlined above also indicate.

The maximum amount of cAMP quantified in the imaging area outside the injection cavity was 0.38 mg (76%), 0.36 mg (72%), 0.32 mg (64%) after 10–12 h, and 0.19 mg (33%) after 20 h for the PLGA (75:25) implant, the Pluronic F127 hydrogel, the aqueous solution, and the PLGA (50:50) implant, respectively. The difference with respect to amount of cAMP in the image area is a result of the sustained release of the PLGA (50:50) implant. After 10–12 h, an apparent decrease in cAMP was seen for the PLGA (75:25) and the Pluronic F127 formulations as well as the aqueous solution that can be explained by cAMP diffusing out of the image area. The root-mean-square distance traveled by a cAMP molecule in 12 h is 9 mm, calculated using the diffusion coefficient for cAMP in 0.5% (*w*/*v*) agarose gel (Supplementary Materials Section S1) and the Einstein–Smoluchowski equation. cAMP diffusion out of the image area is a limitation of the current setup and is related to the fact that the area of the release cell (1071 mm^2^) was larger than the image area (588 mm^2^). After 20 h, the release experiments were terminated, and the amount of cAMP in the agarose gel and formulation in the cavity were quantified by HPLC. The recovered amounts of cAMP were 89 ± 3% (83 ± 3% in the gel) and 94 ± 2% (88 ± 2% in the gel) relative to the injected amount for release experiments conducted with the aqueous solution and the PLGA (75:25) implant, respectively. The obtained release profiles (Figure 5) as well as the results from the HPLC analysis indicate high repeatability of the in vitro testing setup.

Somewhat surprisingly, the release profiles obtained for the aqueous cAMP solution and the Pluronic F127 hydrogel were initially very similar. To facilitate interpretation of the obtained release profiles, the cAMP diffusion coefficients in 20% (*w*/*w*) Pluronic F127 gel, 0.5% (*w*/*v*) agarose gel, and aqueous solution were determined (Supplementary Materials Section S1, including Figures S8 and S9). The cAMP diffusivity in Pluronic F127 gel (9.3 × 10^−10^ m^2^ s^−1^ by UV imaging) was lower as compared to the agarose gel (3.6 × 10^−10^ m^2^ s^−1^ by UV imaging) and aqueous solution (6.9 × 10^−10^ m^2^ s^−1^ by Taylor dispersion analysis). The Pluronic F127 gelation is associated with micelles formed at increasing temperature due to dehydration of the poly-propylene oxide (PPO) blocks [38]. The Pluronic F127–agarose gel distribution coefficient for cAMP is close to one (*K* = 1.21; Supplementary Materials Section S1). This together with the relatively similar diffusion coefficients and the agarose gel being part of the diffusional barrier lead to the almost identical initial release profiles.

#### 2.3.1. Drug Load Study

**Figure 6:**
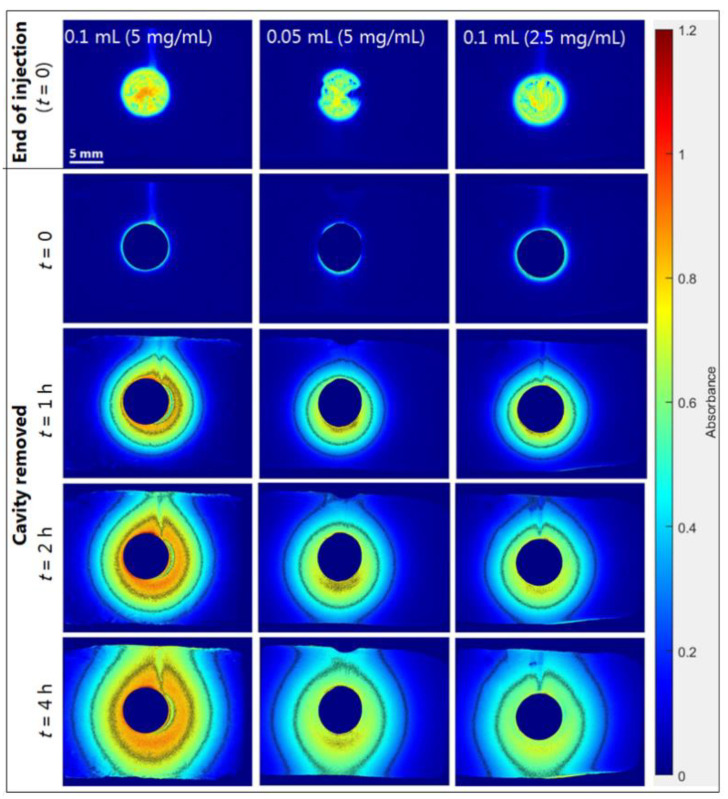
Representative absorbance images obtained at 280 nm showing the release of cAMP from the in situ forming Pluronic F127 hydrogel from 0.1 mL injected (5 mg/mL), 0.05 mL injected (5 mg/mL), and 0.1 mL injected (2.5 mg/mL) into agarose at selected time-points and 37 °C. Contour lines represent absorbance values of 0.25, 0.5, 0.75, and 1. End of injection was the first image recorded, after instillation of the formulation was complete with the needle removed (defined as *t* = 0). Cavity removed referred to absorbance images, where the cavity area was excluded by multiplying with the auto-generated binary image. Note: The absorbance images shown for the Pluronic F127 hydrogel (0.1 mL (5 mg/mL)) are identical to those shown in Figure 4.

**Figure 7:**
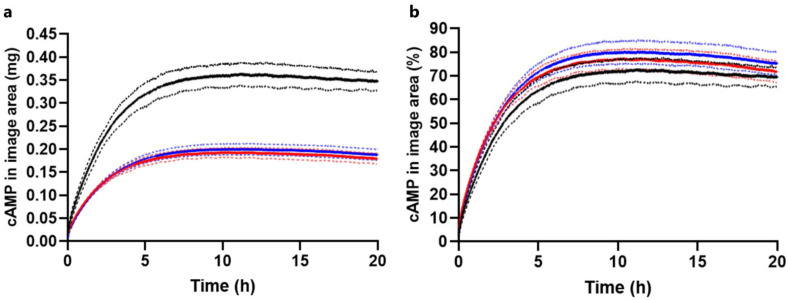
Amount of cAMP in the image area outside the cavity. Apparent release profiles for the in situ forming Pluronic F127 hydrogel. Amounts of 0.1 mL (5 mg/mL) injected (black), 0.05 mL (5 mg/mL) injected (blue), and 0.1 mL (2.5 mg/mL) injected (red) at 37 °C. (**a**) Absolute amount of cAMP. (**b**) Percentage cAMP relative to injected amount. Average of 3 replicates are in bold and error bars (1 SD) are represented by the dotted lines. Note: The apparent release profile shown for the 0.1 mL injected (5 mg/mL) is identical to the profile shown in Figure 5.

Release experiments with different drug loads were conducted with the in situ forming Pluronic F127 hydrogel (Section 3.3). Figure 6 shows selected absorbance images, and Figure 7 shows the corresponding cAMP release profiles obtained upon injection of 0.1 mL (5 mg/mL), 0.05 mL (5 mg/mL), and 0.1 mL (2.5 mg/mL) of the in situ forming Pluronic F127 hydrogel. For the experiments where a cAMP dose of 0.25 mg was injected, the absorbance images were similar and the absorbance values were clearly lower as compared to those observed when injecting 0.5 mg cAMP (Figure 6). In Figure 7a, the difference in drug load is evident, as the release profiles for 0.05 mL (5 mg/mL) and 0.1 mL (2.5 mg/mL) injected had a maximum of 0.20 mg, while the release profiles for 0.1 mL (5 mg/mL) had a maximum of 0.36 mg. The results indicate that the drug load did not affect release rate in percentage (Figure 7b). The recovered amounts of cAMP in the cell relative to the injected amounts were quantified by HPLC and were 96 ± 1% (89 ± 1% in the gel) and 100 ± 4% (94 ± 1% in the gel) for release experiments conducted with the Pluronic F127 hydrogel at 0.1 mL (2.5 mg/mL) and 0.05 mL (5 mg/mL), respectively.

#### 2.3.2. Effect of Formulation Geometry on Release

**Figure 8:**
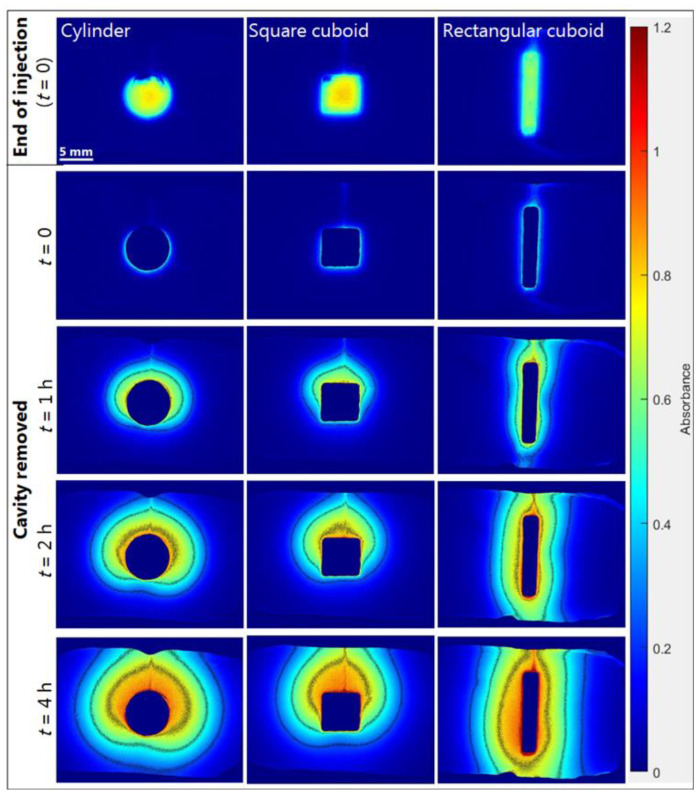
Representative absorbance images showing the release of cAMP (PLGA (75:25) implant) from a cylinder (3.1 × 4 mm^2^ (r × H)), square cuboid (5.5 × 5.5 × 4 mm^3^ (L × W × H)), and rectangular cuboid (12 × 2.5 × 4 mm^3^ (L × W × H)) into agarose at 37 °C. Contour lines represent absorbance values of 0.25, 0.5, 0.75, and 1. End of injection was the first image recorded, after instillation of the formulation was complete with the needle removed (defined as *t* = 0). Cavity removed referred to absorbance images, where the cavity area was excluded by multiplying with the auto-generated binary image. Note: The absorbance images shown for the cylinder are identical to those shown in Figure 4.

**Figure 9:**
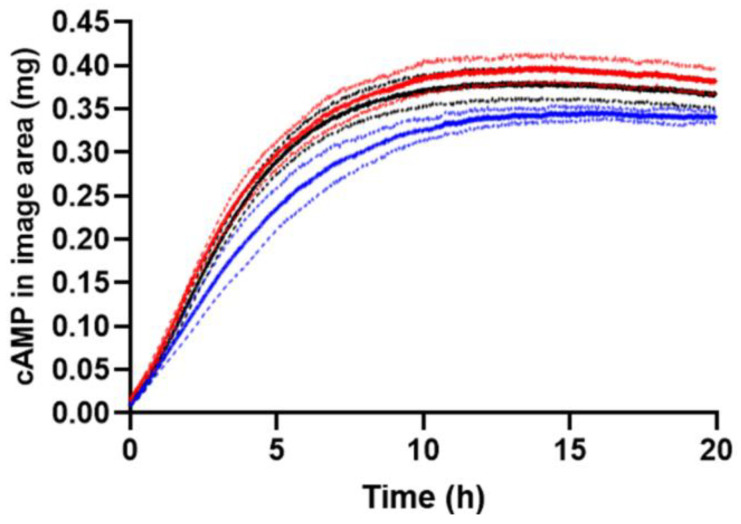
Amount of cAMP in the image area outside the cavity. Apparent release profiles for the formulation geometry studies with the PLGA (75:25) implant. Cylinder (black), square cuboid (blue), and rectangular cuboid (red) at 37 °C. Average of 3 replicates are in bold, and error bars (1 SD) are represented by the dotted lines. Note: The apparent release profile shown for the cylinder is identical to the profile shown in Figure 5.

Ideally, injections should be made directly into the matrix, as this to a larger extent mimics the in vivo conditions. However, defining the geometry of in situ forming injectables in in vitro release studies is currently a hot topic [39,40,41], as it may be advantageous for certain formulation types and for reducing between-experiment variability. To study the impact of the geometrical shape of formulations on the diffusion patterns and the rate of cAMP release, experiments were conducted with different cavity geometries (described in Section 3.2). The in situ forming PLGA (75:25) implant and the Pluronic F127 hydrogel were used for these studies. The geometries had the same volume (0.12 mL/120 mm^3^) but different formulation–gel interface areas. The formulation–gel interface area was 80, 88, and 116 mm^2^ for the cylinder, square cuboid, and rectangular cuboid, respectively. The larger formulation–gel interface area for the rectangular cuboid might result in a faster release of cAMP as compared to formulations placed in the cylinder and the square cuboid. Selected absorbance images in Figure 8 and Figure S6 (Supplementary Materials) show that the transport pattern for the rectangular cuboid was different from those of the cylinder and square cuboid for the PLGA (75:25) and Pluronic F127 formulations. Depot formation was monitored at 520 nm without interference from cAMP and showed that the PLGA (75:25) depot was evenly distributed in the square cuboid and rectangular cuboid, while in the cylinder, the depot mainly resided at the bottom of the cavity (Supplementary Materials Figure S5). For the Pluronic F127 hydrogel, the release of cAMP did not follow the upward oriented transport pattern for the cylinder and the square cuboid geometry (Supplementary Materials Figure S6). Thus, the special concentration distribution observed for the PLGA (75:25) implant might be due to the in situ formation process involving NMP efflux and water influx. From Figure 9, it appears that the initial rate of cAMP release from the square cuboid was slower compared to the release from the cylinder and rectangular cuboid for the PLGA (75:25) implant. However, the recovered amount of cAMP at the end of the experiment was similar for all three geometrical shapes. The reason for the slower cAMP release for the square cuboid geometry is unknown. The amounts of cAMP recovered in the cell relative to the injected amount as determined by HPLC were 94 ± 2% (88 ± 2% in the gel), 95 ± 1% (91 ± 1% in the gel), and 94 ± 2% (89 ± 2% in the gel) for PLGA (75:25) implant release experiments conducted with the cylinder, the rectangular cuboid, and the square cuboid, respectively. For the Pluronic F127 hydrogel, the release profiles were independent of the geometrical shapes (data not shown). The results demonstrate the ability to control shape (geometry) attained for in situ forming formulations upon injection. The cylindrical cavity was the preferred default configuration, as it ensured symmetric diffusion for early time-points. In relation to the in vivo situation, selection of geometry may be less straightforward. In future studies, the geometry may be optimized for selected formulations or injection sites. For instance, relatively thin elongated shapes may be of particular relevance for intra-muscular injection [42] in order to mimic formulation spread between muscle fibers.

## 3. Materials and Methods

### 3.1. Materials

Adenosine 3′,5′-cyclic monophosphate (cAMP), sodium adenosine 3′,5′-cyclic monophosphate monohydrate (NacAMP × H_2_O), agarose (type 1, low EEO), Na_2_HPO_4_ × 2H_2_O, 1-methyl-2-pyrrolidinone (NMP), and PLGA (RESOMER^®^ RG 503 with lactide:glucolide molar ratio 50:50, ester terminated, molecular weight 24–38 kDa) were obtained from Sigma-Aldrich (St. Louis, MO, USA). NaH_2_PO_4_ × H_2_O and PLGA (EXPANSORB^®^ DLG75 2A with lactide:glycolide molar ratio of 75:25, acid terminated, molecular weight 5–20 kDa) were obtained from Merck Emsure (Darmstadt, Germany). Pluronic F127 (PEO_99_–PPO_67_–PEO_99_) was a gift from BASF (Mount Olive, NJ, USA). Purified water from an SG Ultra Clear^TM^ 2002 water system (SG Water GmbH, Barsbüttel, Germany) was used for the preparation of buffer and formulations. The phosphate buffer used was 67 mM, pH 7.40 (*I* = 0.17 M). Agarose gel was made by weighing agarose corresponding to 0.5% (*w*/*v*) suspended in buffer and heated at 95 °C using a water bath until dissolved; upon cooling the gel was formed.

#### Preparation of cAMP Formulations

Four formulations containing cAMP were made. An aqueous solution of cAMP in 67 mM phosphate buffer, pH 7.40, was prepared by weighing cAMP and dissolving it in a volume corresponding to 5 mg/mL (1.5 × 10^−2^ M). The in situ forming Pluronic F127 hydrogels loaded with 5 mg/mL or 2.5 mg/mL cAMP were prepared using the cold method [43]. The polymer was dissolved in phosphate buffer corresponding to 20% (*w*/*w*) at 5 °C under stirring. A weighed amount of cAMP was dissolved in a measured volume of cold 20% (*w*/*w*) Pluronic F127 solution. Two in situ forming PLGA formulations were prepared with different grades and polymer concentrations. An in situ forming PLGA (75:25) formulation was prepared by dissolving PLGA (75:25) in NMP corresponding to 40% (*w*/*w*) and left under stirring at room temperature until a clear solution was obtained. NacAMP was weighed, and a measured volume of the PLGA-NMP solution was added to achieve a 40% (*w*/*w*) PLGA-NMP solution loaded with NacAMP corresponding to 5 mg/mL cAMP. An additional in situ forming PLGA (50:50) formulation was prepared by dissolving PLGA (50:50) in NMP corresponding to 33% (*w*/*w*) and left under stirring at room temperature until a clear solution was obtained. NacAMP was weighed and added corresponding to 0.5% (*w*/*w*) cAMP (5.6 mg/mL).

### 3.2. UV Imaging System

An SDi2 UV–Vis imaging system (Pion Inc., Billerica, MA, USA) and a custom-made 3D-printed release cell were used for imaging the release experiments. The SDi2 has a detection area (CMOS chip) of 28 × 28 mm^2^ (2048 × 2048 pixels) and a single-pixel size of 5.5 µm with 2.5:1 magnification (effective pixel size 13.75 µm). The SDi2 is capable of dual-wavelength imaging with four available wavelengths in the UV range (255, 280, 300, and 320 nm ± 5 nm) and one in the visible range (520 nm ± 5 nm) [44]. The 3D-printed release cell (Ultimaker 3 extended, Ultimaker, Geldermasse, Netherlands) made from PLA with inner dimensions of 30 × 21 × 4 mm^3^ (*L* × *W* × *H*) and an injection channel with a diameter of 1.2 mm was used for the release experiments (Figure 1). Two quartz slides (AdValue Technology, Tuscon, AZ, USA) with dimensions 75 × 26 × 1.0 mm^3^ (*L* × *W* × *D*) were used as the top and bottom layers of the 3D-printed release cell. The release cell with the two quartz slides was placed in a metal casing, allowing it to be mounted in the SDi2. For the diffusion and distribution experiments, diffusion cells (quartz cuvettes) with inner dimensions of 38.0 × 8.0 × 1.0 mm^3^ (*L* × *W* × *H*; Starna Scientific Ltd., Hainnault, Essex, UK), cuvette mounts (Hellma Analytics, Baden-Württemberg, Germany), and a 3D-printed cuvette holder were used (printed using a MakerBot Replicator 2, MakerBot Industries, New York, NY, USA; Supplementary Materials Figure S7). The experiments were conducted at 37 ± 1 °C. A metal cylinder (3.1 × 4 mm^2^ (*r* × *H*)) was used for defining the geometry of the cavity. A square cuboid (5.5 × 5.5 × 4 mm^3^ (*L* × *W* × *H*)) and a rectangular cuboid (12 × 2.5 × 4 mm^3^ (*L* × *W* × *H*)) were 3D-printed (MakerBot Replicator 2, MakerBot Industries, New York, NY, USA) and used for the formulation geometry study.

The 3D-printed release cell was used for the release experiments using the following approach. The geometries for defining the cavity were placed at the center of the release cell, and approximately 4.4 mL agarose solution (0.5% (*w*/*v*)) heated to 95 °C was transferred to the release cell, without the top quartz plate, until slightly overfilled (Figure 1a, *V*_cell_ = 4.3 mL). The geometries were removed after 15 min, thereby creating cavities in the agarose gel. The cavity was filled with buffer, and the top quartz plate was placed on top. The assembled release cell was placed in a metal casing and mounted in the SDi2 (Figure 1).

### 3.3. UV–Vis Imaging Release Experiments

The UV imaging setup is schematically shown in Figure 1c. UV imaging was performed using the SDi2, the 3D-printed release cell, and a syringe pump (Fusion 200, Chemyx Inc., Stafford, TX, USA), which was placed outside the SDi2. Sirius SDi2 Collection software v.1.2.0 (Pion Inc., Billerica, MA, USA) was used for recording the images. After referencing, the recording was paused, and the needle was inserted into the cavity. The recording was resumed, and cAMP-loaded formulations were injected. The drug release process was followed for 20 h, applying a frame rate of 1 frame/5 s for the first min and thereafter 1 frame/50 s. The wavelengths used were 280 nm and 520 nm. The temperature was measured during release experiments by inserting a probe (Fluke 3000, Fluke Corporation, Everett, WA, USA)) into one of the additional outlets of the release cell (Figure 1b). The formulations were injected using a syringe pump (0.1 mL at 0.2 mL/min) and a 1 mL syringe (Chirana T. Injecta, Stara Tura, Slovakia). The syringe was connected to a hypodermic needle via a polypropylene tube (i.d. 1 mm). A 27G (0.4 mm OD) was used for injection of the aqueous solution and the in situ forming 40% (*w*/*w*) PLGA (75:25) formulation, whereas a 20G (0.9 mm OD) needle was applied for of the in situ forming Pluronic F127 hydrogel. The in situ forming 33% (*w*/*w*) PLGA (50:50) formulation was injected manually due to a high viscosity using a 20G needle. Release experiments where the drug load was decreased by 50% (0.25 mg cAMP) were conducted by injecting either a smaller volume of the Pluronic F127 formulation or a Pluronic F127 formulation containing 2.5 mg/mL of cAMP. The effect of formulation geometry was studied by using the 3D-printed geometries (square cuboid and rectangular cuboid) as described in Section 3.2. The formulation used for these experiments was the in situ forming 40% (*w*/*w*) PLGA (75:25) formulation. For selected release experiments, the agarose gel and the formulation in the cavity were separated and recovered at the end of the experiment for quantitation of cAMP using HPLC.

#### HPLC Analysis

Reverse-phase HPLC was applied for quantification of cAMP in the agarose gel and in the formulation using a Gemini C18 column (150 × 4.6 mm^2^; 5 µm; Phenomenex Inc., Torrance, CA, USA) with a Gemini C18 precolumn (4 × 3.0 mm^2^, Phenomenex, Torrance, CA, US). A YL9100 HPLC system consisting of an YL9110 quaternary pump, an YL9101 vacuum degasser, an YL9130 column compartment, an YL9160 PDA detector, and an YL9150 autosampler (YL Instruments Co., Ltd., Gyeonggi-do, Korea) was applied for the HPLC analysis. The mobile phase consisted of methanol and 20 mM phosphate buffer (pH 6.6) in the ratio of 10:90 (*v*/*v*) for release experiments with the 40% (*w*/*w*) in situ forming PLGA (75:25) formulation and 15:85 (*v*/*v*) methanol: phosphate buffer for the in situ forming Pluronic F127 hydrogel and the aqueous solution. The injection volume was 20 µL, the flow rate was 1 mL/min, and the effluent was monitored at 259 nm at 30 °C. Calibration curves (R^2^ > 0.99) were made for cAMP in the concentration range of 3.20 × 10^−6^–3.80 × 10^−5^ M. Samples were prepared by suspending the recovered agarose gel in 67 mM phosphate buffer. The formulation present in the cavity was suspended in 67 mM phosphate buffer (aqueous solution and Pluronic F127 hydrogel) or NMP (PLGA (75:25) implant). The agarose gel suspended in buffer was heated to 95 °C for 1 h and centrifuged before the supernatant was analyzed. The formulations in buffer were analyzed directly, while the PLGA implant dissolved in NMP was transferred to 67 mM phosphate buffer before it was centrifuged and the supernatant analyzed.

### 3.4. Instrument Performance and Image Analysis

#### 3.4.1. Determination of Resolution

Grids consisting of a black image made of silver halides (5 µm printer layer thickness) coated on one side of a 180 µm polyester plastic base were used to assess the resolution of the SDi2 system as described previously [33]. The width of the lines varied in the range of 10–100 µm in 10 µm increments. Measurements were performed at 520 nm, where the plastic base was transparent and the grids absorbed light. The resolution was determined from the ability to resolve line pairs by the imaging system when the grids were placed directly on the CMOS chip and in front of the assembled release cell filled with 0.5% (*w*/*v*) agarose gel (12 mm distance to CMOS chip).

#### 3.4.2. Calibration Curves

Calibration curves of cAMP in the concentration range of 6.25 × 10^−5^–4 × 10^−3^ M (2.1 × 10^−2^–1.3 mg/mL) were constructed in the release cell (light path of 4 mm) and in diffusion cells (light path of 1 mm) at 37 °C. The absorbance of cAMP at 280 nm in 0.5% (*w*/*v*) agarose gel was determined for the release cell as well as in 0.5% (*w*/*v*) agarose gel and 20% (*w*/*w*) Pluronic F127 hydrogel for the diffusion cell. For the recording of reference images, the respective medium without cAMP was used. Absorbance values were determined using Sirius SDi2 Data Analysis (v. 3.0.20, Pion Inc., Billerica, MA, USA).

The calibration curves were fitted to Equation (1) using the least-square fit in GraphPad Prism 8 (v. 8.4.3, GraphPad Software, San Diego, CA, USA):

(1) $$A=A_{\max}-\left(A_{\max}-A_{0}\right)\cdot e^{-kC}$$

where *A*_max_ is the plateau value, *A*_0_ is the y-intercept, *C* is the concentration, and *k* is a constant of the exponential plateau function.

#### 3.4.3. Image Analysis of Release Experiments

Image files obtained from the release experiments were converted to TIF images (grayscale intensity) using D100 image viewer and editor (v. 1.0, Paraytec Ltd., York, UK). The images were analyzed using MATLAB R2019a (v. 9.6.0.1472908, Mathworks Inc., Natick, MA, USA). Imported images in MATLAB were treated as a matrix of pixel intensities. Absorbance was calculated from light intensity and corrected for stray light and electronic noise using [45]:

(2) $$A=\log\left(\frac{P_{0}-P_{s}}{P-P_{s}}\right),$$

where *P*_0_, *P*_s_, and *P* are the pixel intensities collected for the reference, dark, and sample images, respectively. The cavity area and the edges of the release cell were removed by multiplying with an auto-generated binary image. Absorbance images constructed using Equation (2) were used to calculate the concentration of cAMP for each pixel using the calibration curve for cAMP in the agarose gel. The concentration of cAMP was converted to the amount of cAMP by multiplying with the volume of each pixel given by the dimensions (13.75 × 13.75 × 4000 µm^3^). The values for the individual pixels were summed to achieve the total amount of cAMP in the imaging area. The amount of cAMP in the image area was plotted against the corresponding time-points for all images (0–20 h) using a conventional for-loop function. The image files were sorted using a natural order file name sort function from the MATLAB Central File Exchange database [46]. A color scale and contour lines were added to the absorbance images at selected time-points to visualize the diffusion patterns.

### 3.5. cAMP Diffusion and Distribution in Agarose and Pluronic F127 Gels

Diffusion studies (*n* = 3) were conducted to determine the diffusion coefficient of cAMP in 0.5% (*w*/*v*) agarose gel and 20% (*w*/*w*) Pluronic F127 gel at 37 °C. The experiments were conducted in diffusion cells (1 mm quartz cuvettes) using a 3D-printed holder and cuvette mounts (Supplementary Materials Figure S7a). The diffusion cells were filled with either blank agarose gel or blank Pluronic F127 gel, and referencing was performed upon settling of the gel. A horizontal cut in the middle was made, and half of the gel was removed. Agarose or Pluronic F127 gel containing 2 × 10^−4^ M cAMP were transferred to the empty half of the cuvette and placed in the SDi2 for six hours. The diffusion of cAMP from the donor compartment (top) to the acceptor compartment (bottom) was determined by measuring the absorbance at 280 nm at selected time points. A distribution study (*n* = 3) using this setup was also conducted to achieve the distribution coefficient for cAMP between Pluronic F127 and agarose gel. Figure S7b (Supplementary Materials) shows a schematic of the experimental configuration.

### 3.6. Diffusion and Distribution Analysis

The spatially and temporally resolved UV absorbance maps were converted to the corresponding concentration maps using the cAMP calibration curves. Normalized concentration–distance profiles at selected time-points were fitted to Equation (3) to achieve the diffusion coefficient (*D*) for cAMP in 0.5% (*w*/*v*) agarose and 20% (*w*/*w*) Pluronic F127 gel [19,47].

(3) $$\frac{C\left(x,t\right)}{C_{0}}=\frac{1}{2}-\frac{1}{2}erf\left(\frac{x-x_{0}}{2\sqrt{Dt}}\right),$$

where *C*_0_ is the initial cAMP concentration in the donor compartment, *C(x,t)* is the concentration of cAMP in the gel at the distance *x* at time *t*, and *x*_0_ is the position of the interface. Both *D* and *x*_0_ were obtained from the least-square fit using GraphPad Prism 8. Due to an imperfect boundary between the donor and acceptor compartment at *t* = 0, the observed diffusion coefficient was dependent on the diffusion time and was corrected by plotting the observed diffusion coefficients as a function of the reciprocal time. The corrected diffusion coefficient was obtained as the y-intercept for this plot [19].

For cAMP diffusing in a system comprising two different phases, such as from Pluronic F127 gel to agarose gel, the flux of cAMP molecules in the Pluronic F127 gel and the agarose gel is equal to:

(4) $$D_{F127}\frac{\partial C_{F127}}{\partial x}=D_{\mathrm{Aga}}\frac{\partial C_{\mathrm{Aga}}}{\partial x},$$

where *C*_F127_, *C*_Aga_, *D*_F127_, and *D*_Aga_ are the concentrations of cAMP in Pluronic F127 and agarose gel and the diffusivities of cAMP in Pluronic F127 and agarose gel, respectively, and *x* is the distance from the interface between the two compartments. The cAMP concentration was equal to *C*_0_ and zero in the Pluronic F127 gel compartment and the agarose gel compartment, respectively, at *t* = 0. It was assumed that the cAMP concentration at the ends of the cell was zero for the agarose gel and *C*_0_ for the Pluronic F127 gel throughout the experiment. The following equations were used to determine the distribution coefficient for cAMP using the least-square fit in GraphPad Prism 8. [47,48]:

(5) $$C_{F127}\left(x,t\right)=C_{0}-\frac{C_{0}}{1+K\sqrt{D_{F127}/D_{\mathrm{Aga}}}}erfc\left(\frac{-x}{2\sqrt{D_{F127}t}}\right),\;\;\;-\infty <x<0,$$

(6) $$C_{\mathrm{Aga}}\left(x,t\right)=\frac{C_{0}\sqrt{D_{F127}/D_{\mathrm{Aga}}\;}}{1+K\sqrt{D_{F127}/D_{\mathrm{Aga}}}}erfc\left(\frac{x}{2\sqrt{D_{\mathrm{Aga}}t}}\right),\;\;\;\;\;\;\;\;\;0<x<\infty ,$$

where *K* is the distribution constant for cAMP between the two phases.

### 3.7. Taylor Dispersion Analysis (TDA)

Taylor dispersion analysis was conducted using a Beckman P/ACE MDQ equipped with a diode array detector (Beckman-Coulter, Fullerton, CA, USA). The UV traces were recorded with the Beckman 32-Karat software 7.0 (Beckman) at a wavelength of 214 nm. A fused silica capillary (Polymicro Technologies, Phoenix, AZ, USA) with a total length of 50.2 cm (75 µm id) and 40 cm to the detection window was used for the dispersion measurements. Samples containing 2 × 10^−4^ M cAMP in 0.067 M phosphate buffer, pH 7.4, were introduced into the capillary by applying a pressure of 0.5 psi for 3 s into the capillary filled with the buffer, followed by a mobilization pressure of 0.7 psi to force the sample plug through the capillary with buffer (*n* = 7). The temperatures of the sample tray and capillary cartridge were set to 25 °C and 37 °C, respectively. The diffusion coefficient of cAMP was determined using Fida data analysis software (Fida Biosystems ApS, Søborg, Denmark).

## 4. Conclusions

The UV imaging release-testing method intended for intra-tumoral injectables constitutes the first setup for characterization of injectable formulations compatible with the SDi2 system. The static setup comprised a UV transparent agarose gel as a model for the tumorous tissue and was found to be effective for monitoring the early events occurring during and immediately after the instillation of the formulations.

The study led to new insights on UV imaging performance. The effective resolution of the SDi2 instrumentation is in the range of 30 µm to 80 µm, making the system suited to monitoring drug transport, i.e., diffusion, in the release setup. The resolution, however, does not allow for detection of, for instance, nanoparticles. The linear range of the SDi2 is less than that of most traditional spectrophotometers. This was addressed by applying non-linear calibration curves. The background absorbance of the matrix affected to a large extent the curvature (deviation from Beer’s law) of the cAMP calibration curves. The developed MATLAB scripts enabled flexible data analysis with respect to definition and utilization of quantification zones, absorbance maps with contour lines improving visualizing of transport patterns, and continuous analysis facilitating the analysis of all images within a series (1500 images recorded over 20 h).

The in vitro release of cAMP was assessed using a UV imaging approach. The aqueous solution, the in situ forming Pluronic F127 hydrogel, and the in situ forming PLGA (75:25) implant exhibited relatively similar initial cAMP release rates. More sustained cAMP release was obtained from the in situ forming PLGA (50:50) implant. The prototype setup was effective in visualizing effects of depot geometry (cylinder, square cuboid, or a rectangular cuboid), and the absorbance maps showed differences in diffusion patterns. 

The prototype UV imaging release setup combined with MATLAB data analysis constitutes a significant technical development in terms of visualizing and characterizing parenteral formulation behavior upon introduction into a biomimetic matrix. In vivo validation should be pursued, and additional studies may be directed at increasing biorelevance by incorporating extracellular matrix components.

## Data Availability

The data presented in this study are available on reasonable request from the corresponding author.

## Supplementary Materials

The following are available online at https://www.mdpi.com/article/10.3390/ijms23073599/s1.
