# Supplementary material for: Methodological Considerations in Development of UV Imaging for Characterization of Intra-Tumoral Injectables Using cAMP as a Model Substance
Source: Int J Mol Sci. 2022 Mar 25;23(7):3599. doi: 10.3390/ijms23073599 (PMC8998202; doi:10.3390/ijms23073599)
Supplement: Supplementary file 1 [file ijms-23-03599-s001.zip › ijms-1602498-supplementary.pdf]

Supplementary Material

Methodological considerations in development of UV imaging for characterization of intra-tumoral injectables using cAMP as a model substance

Frederik Bock [1], Johan Peter Bøtker [1], Susan Weng Larsen [1], Xujin Lu [2] and Jesper Østergaard [1,*]

[1] Department of Pharmacy Faculty of Health and Medical Sciences, University of Copenhagen, Universitetsparken 2, DK-2100 Copenhagen, Denmark; frederik.bock@sund.ku.dk (F.B.); johan.botker@sund.ku.dk (J.B.); susan.larsen@sund.ku.dk (S.L.)
[2] Bristol Myers Squibb Company, Drug Product Development, 1 Squibb Drive, New Brunswick, 08901 New Jersey, USA; xujin.lu@bms.com (X.L.)
* Correspondence: jesper.ostergaard@sund.ku.dk (J.Ø.); Tel.: +4535336138

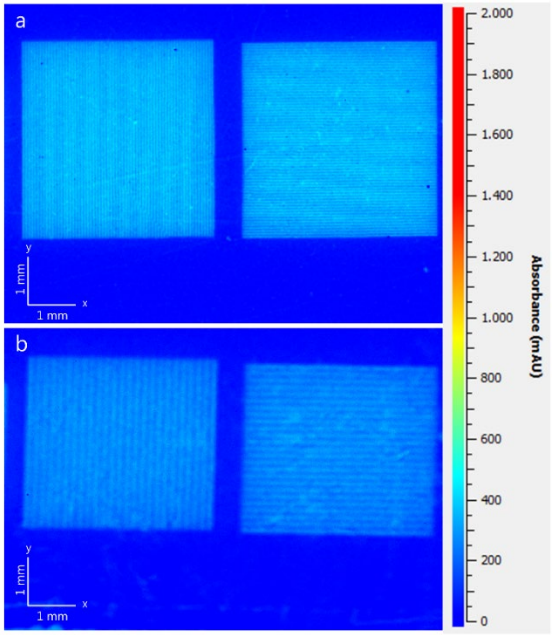

Figure S1. Absorbance maps of grids at different distance to the CMOS sensor obtained at 520 nm. (**a**) 30 μm line grid placed directly on the CMOS sensor (optimal conditions). (**b**) 80 μm line grid placed in front of the release cell filled with 0.5% (w/v) agarose gel (least optimal conditions, 12 mm from CMOS chip).

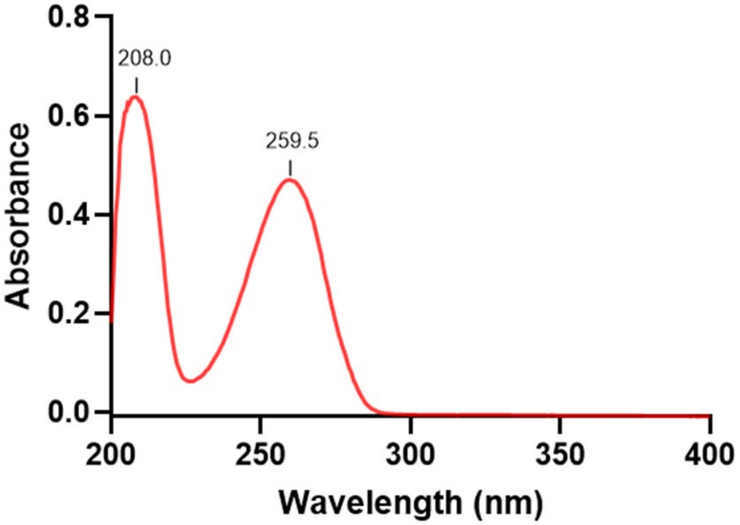

Figure S2. UV spectrum of $2.5 \cdot 10^{-4}$ M cAMP in 67 mM phosphate buffer (10 mm light path cuvette).

```matlab
%% Reference images
clear all; close all; clc;
%import Dark images (stray light)
Counter = 1;
Dc = [];
for i =[4500 5500 6500 7500 8500 9500 10500 11500 12500 13500]; %timestamp for dark images (n=10)
    Dc(:,:,Counter) = im2double(imread([num2str(i) '.tif'])); %store the images as a 10 sided dice
    Counter = Counter + 1;
end
D = mean(Dc,3);

%Reference intensity images (I0)
Counter = 1;
I0c = [];
for i =[20500 21500 22500 23500 24500 25500 26500 27500 28500 29500]; %timestamp for I0 images (n=10)
     I0c(:,:,Counter) = im2double(imread([num2str(i) '.tif']));%store the images as a 10 sided dice
     Counter = Counter + 1;
end
I0 = mean(I0c,3);

R = I0-D;% Reference image

%% Segmentation - binarizing and resmoothing
%Reversing binary R image - Used to remove absorbance inside cavity
IV = im2double(uint8(255))- R ;
BWC = imbinarize(IV,0.30); %Threshold value is adjustable

%Resmoothing of reverse binary cavity image
windowSize = 50;
kernel = ones(windowSize) / windowSize ^ 2;
blurryImage = conv2(single(BWC), kernel, 'same');
BWC = blurryImage > 0.5;

%Removing egdes around the gel
BWR = imbinarize(R);
windowSize = 50;
kernel = ones(windowSize) / windowSize ^ 2;
blurryImage = conv2(single(BWR), kernel, 'same');
BWR = blurryImage > 0.5;

BW = BWC.*BWR; %Binary image - zero removed from the analysis

%% pixel count
BW = BWC.*BWR;
ntotal = numel(BW);
nwhite = sum(BW(:));
Image_area = nwhite.*13.75^2.*10^-6;


Cavity = imcrop(BWC,[200 200 1448 1248]);

nblack = sum(Cavity(:) == 0);

Cavity_Area = nblack.*13.75^2.*10^-6;

%% Absorbance and Amount calculation - For loop
path = fileparts(pwd); %Goes one folder back
projectdir = uigetdir(path); %select folder with images to be analyzed
S = dir(fullfile(projectdir, '*.tif'));
[~,ndx] = natsortfiles({S.name}); %Sorts the imagename (timestamp) in the natural order (MATLAB exchange)
S = (S(ndx)); %struct of images sorted by timestamp
numfiles = length(S); %how many images that are in the frames folder

parfor i = 1 : numfiles
    filename = fullfile(projectdir, S(i).name);
    thisimage = im2double(imread(filename)); %Import of image and conversion to double format
    Abs = real((log10(R./(thisimage-D)))); %Equation for converting intensity to absorbance
    Abs(isnan(Abs))=0; %removal of Nan values
    Abs (Abs >1.174)=1.173; %Max Abs
    Conc = (log(-1.17647./(Abs-1.174))/1378).*BW; %Calibration curve used for converting abs to conc
    Am = ((Conc)*13.75*10^-6*13.75*10^-6*4*10^-3*1000)*329.21; %Amount is calculated using pixel dimensions and molarmass
    Amt=sum(sum(Am)); %The amount for each pixel is summed

    outputsAmt(i,1) = Amt;
end
Cell=struct2cell(S); %conversion of struck to cell for S
Timestamp= str2double(cellfun(@(x) x(1:end-4), Cell(1,:)','UniformOutput', false)); % Removing columns except column 1 and remove .tif from filename
Time_0=Timestamp(1,:);%Time for End of injection
Time = (Timestamp-Time_0)/(60.*60.*1000);%conversion of timestamp to hours
Results = ([Time outputsAmt]);

save('Results', 'Results')
```

Figure S3. MATLAB script for quantification of cAMP in the gel by image analysis.

```matlab
%% Reference images
clear all; close all; clc;
%import Dark images (stray light)
Counter = 1;
Dc = [];
for i =[4500 5500 6500 7500 8500 9500 10500 11500 12500 13500]; %timestamp for dark images (n=10)
    Dc(:,:,Counter) = im2double(imread([num2str(i) '.tif'])); %store the images as a 10 sided dice
    Counter = Counter + 1;
end
D = mean(Dc,3);

%Reference intensity images (I0)
Counter = 1;
I0c = [];
for i =[20500 21500 22500 23500 24500 25500 26500 27500 28500 29500]; %timestamp for I0 images (n=10)
     I0c(:,:,Counter) = im2double(imread([num2str(i) '.tif']));%store the images as a 10 sided dice
     Counter = Counter + 1;
end
I0 = mean(I0c,3);

R = I0-D;% Reference image

%% Segmentation - binarizing and resmoothing
%Reversing binary R image - Used to remove absorbance inside cavity
IV = im2double(uint8(255))- R ;
BWC = imbinarize(IV,0.30);
%Resmoothing of reverse BW cavity image
windowSize = 51;
kernel = ones(windowSize) / windowSize ^ 2;
blurryImage = conv2(single(BWC), kernel, 'same');
BWC = blurryImage > 0.5; % Rethreshold

%Removing egdes around the gel and resmoothing
BWR = imbinarize(R);
windowSize = 51;
kernel = ones(windowSize) / windowSize ^ 2;
blurryImage = conv2(single(BWR), kernel, 'same');
BWR = blurryImage > 0.5; % Rethreshold

%% Folder selection
path = fileparts(pwd); %Goes one folder back
selpath = uigetdir(path); %select folder with images to be analyzed
cd(selpath)%% For loop contour images
S = dir(fullfile(selpath, '*.tif'));
[~,ndx] = natsortfiles({S.name});
numfiles = length(S);
S = (S(ndx));

for i = 1 : numfiles
    if i == 1
    filename = fullfile(selpath, S(i).name);
    thisimage = im2double(imread(filename)); %import image and conversion to double
     Abs = real((log10(R./(thisimage-D)))); %Conversion from intensity to absorbance
     Abs = Abs.*BWR; % Remove egdes
     Abs(isnan(Abs))=0; %remove Nan values
     imagesc(Abs,[0 1.2])%absorbance scale can be changed in the  [].
     colormap(jet(256));
     hold on
     colorbar %color scalebar can be removed by inserting % in front of this line.
     saveas(gcf,['Contourw-' num2str(i)],'jpg');
     hold off
    end

    filename = fullfile(selpath, S(i).name);
    thisimage = im2double(imread(filename)); %import image and conversion to double
     Abs = real((log10(R./(thisimage-D)))); %Conversion from intensity to absorbance
     Abs = Abs.*BWR.*BWC; % Remove egdes
     Abs(isnan(Abs))=0; %remove Nan values
     imagesc(Abs,[0 1.2])%absorbance scale can be changed in the  [].
     colormap(jet(256));
     hold on
     colorbar %color scalebar can be removed by inserting % in front of this line.
     contour(Abs,[0.25, 0.5, 0.75, 1] ,'color','k','linestyle', ':');% change increment values for contour lines her[illegible]
     saveas(gcf,['Contour-' num2str(i)],'jpg');
     hold off

end
```

Figure S4. MATLAB script for generating absorbance images with color scale and contour lines.

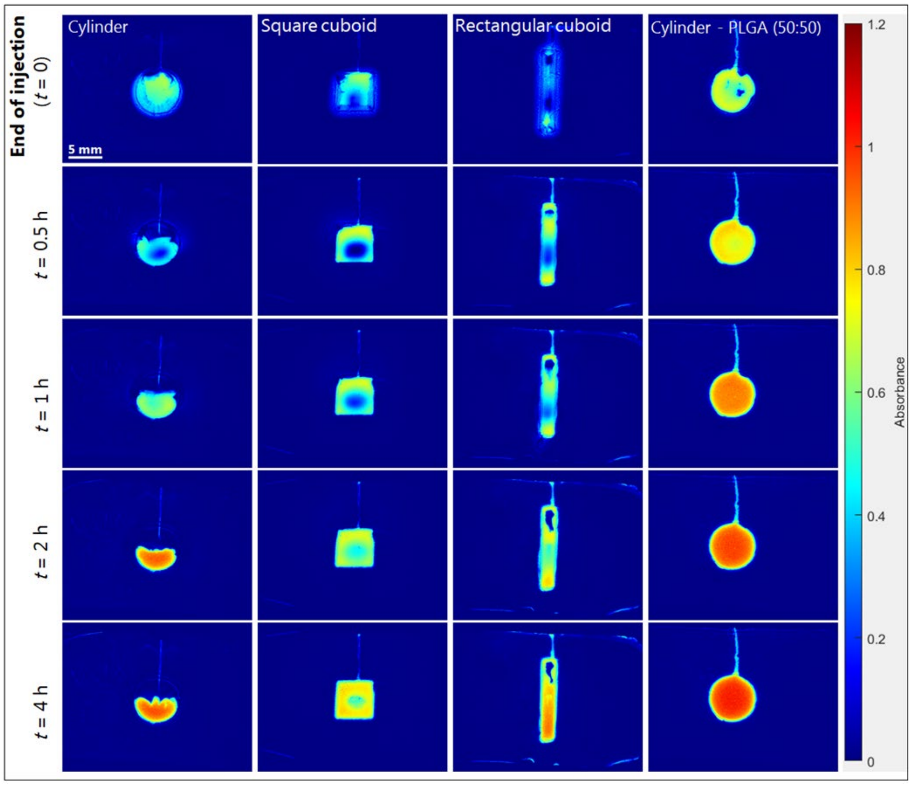

Figure S5. Representative absorbance images at visible wavelength (520 nm) showing the depot formation for a PLGA (75:25) implant after injection into a cylinder (3.1 × 4 mm^2 ($r \times H$)), square cuboid (5.5 × 5.5 × 4 mm^3 ($L \times W \times H$)), rectangular cuboid (2.5 × 12 × 4 mm^3 ($L \times W \times H$)), and a PLGA (50:50) implant after injection into a cylinder (3.1 × 4 mm^2 ($r \times H$)) at 37°C.

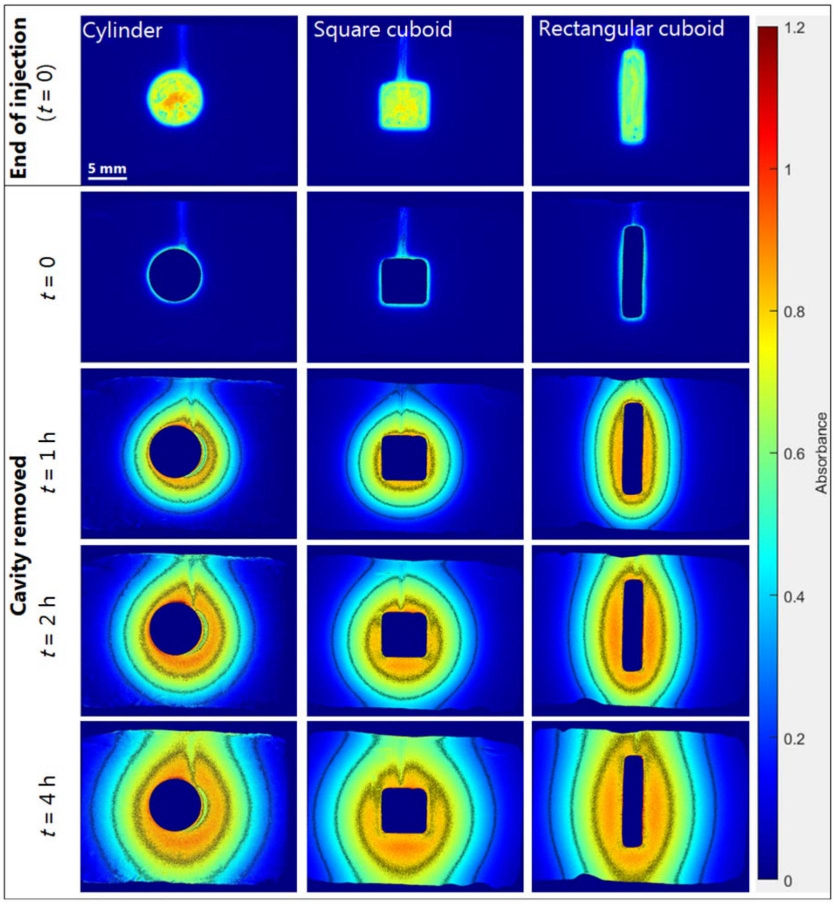

Figure S6. Representative absorbance images showing the release of cAMP (*in situ* forming Pluronic F127 hydrogel, 5 mg/mL cAMP) from a cylinder (3.1 × 4 mm^2 ($r \times H$)), square cuboid (5.5 × 5.5 × 4 mm^3 ($L \times W \times H$)), and rectangular cuboid (12 × 2.5 × 4 mm^3 ($L \times W \times H$)) into agarose at selected time-points and 37°C. Contour lines represent absorbance values of 0.25, 0.5, 0.75, and 1. End of injection was the first image recorded, after instillation of the formulation was complete with the needle removed (defined as t = 0). Cavity removed referred to absorbance images where the cavity area was excluded by multiplying with the auto-generated binary image. Note: The absorbance images for the cylinder are similar to those shown in Figure 4.

1. Diffusion and Distribution Study

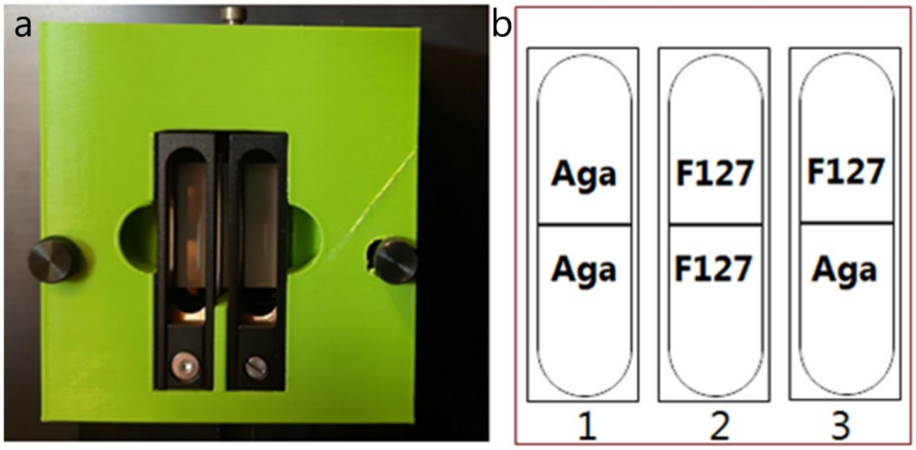

Figure S7. Experimental setup for the diffusion and distribution studies (n = 3). (**a**) 3D-printed cuvette holder containing two cuvette mounts. (**b**) Schematic representation of sample positioning for the diffusion and distribution experiments. Experiment 1 and 2 were diffusion studies (n = 3 for both), while experiment 3 was a distribution study (n = 3). The donor compartment consisted of 0.5% (w/v) agarose gel (Aga) or 20% (w/w) Pluronic F127 gel (F127) containing $2 \cdot 10^{-3}$ M cAMP and was placed at the top of the cuvette. The acceptor compartment without cAMP was added at the bottom.

To facilitate the interpretation of the obtained release profiles, the diffusion of cAMP in 0.5% (w/v) agarose gel and in 20% (w/w) Pluronic F127 gel was investigated. The UV images obtained from the diffusion studies were converted to concentration-distance profiles. The normalized concentration-distance profiles shown in Figure S8a-b were fitted to equation 3 to achieve the diffusion coefficient for cAMP in 0.5% (w/v) agarose gel and 20% (w/w) Pluronic F127 gel. The corrected diffusion coefficient was obtained by plotting the observed diffusion coefficients as a function of the reciprocal time [19] (Figure S9). The corrected diffusivity for cAMP in 0.5% (w/v) agarose gel and in 20% (w/w) Pluronic F127 gel were $9.3 \cdot 10^{-10}$ m^2 s^{-1} (RSD = 7.5%) and $3.6 \cdot 10^{-10}$ m^2 s^{-1} (RSD = 8.3%), respectively. The cAMP diffusion coefficient was lower in 20% (w/w) Pluronic F127 gel as compared to agarose gel. Similar diffusion coefficients in 0.5 % (w/v) agarose gel and aqueous solution ($6.9 \cdot 10^{-10}$ m^2 s^{-1} as determined by Taylor dispersion analysis at 37°C; RSD% = 1.2 %; n = 7) were expected because the pore size in 0.5% agarose gel (600-1200 nm [6]) is considerably larger than the size of small molecules, such as cAMP. The observed difference with respect to diffusivity was considered acceptable due to different methods being applied, TDA and UV imaging. A distribution study using the UV imaging setup (Figure S6) was conducted to determine the distribution coefficient for cAMP between 20% (w/w) Pluronic F127 gel and 0.5% (w/v) agarose gel. The profiles shown in Figure S8c were fitted to equation 5 and equation 6 simultaneously to determine the distribution coefficient (K). The diffusion coefficient in 0.5% (w/v) agarose gel and in 20% (w/w) Pluronic F127 gel were fixed to the corrected diffusion coefficients determined in Figure S8 as it was not possible to achieve appropriate fits with three variables in Graphpad Prism 8. The Pluronic F127 - agarose gel distribution coefficient for cAMP was determined to 1.21 (RSD = 4.5%), and indicated a slightly higher affinity of cAMP for Pluronic F127 gel as compared to the agarose gel.

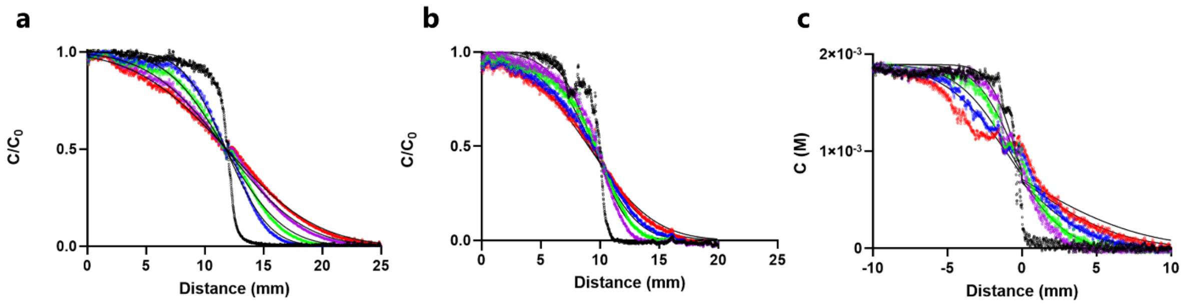

Figure S8. Diffusion and distribution of cAMP in agarose gel and Pluronic F127 gel at 37°C. The donor compartment had a cAMP concentration of $2 \cdot 10^{-3}$ M. (**a**) Normalized concentration-distance profiles for diffusion of cAMP in 0.5% (w/v) agarose gel. (**b**) Normalized concentration-distance profiles for diffusion of cAMP in 20% (w/w) Pluronic F127 gel. (**c**) Concentration-distance profile for the diffusion and distribution between 20% (w/w) Pluronic F127 gel and 0.5% (w/v) agarose gel. t = 0 (●), 1 h (●), 2 h (●), 4 h (●), and 6 h (●). The lines represent the least-squares fit ($R^2 \geq 0.96$) of the concentration-distance profiles to equation 3 (**a** and **b**) or simultaneous fitting to equation 5 and equation 6 (**c**).

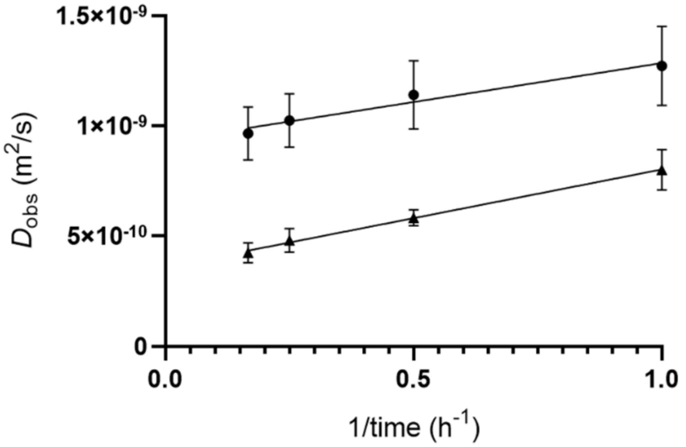

Figure S9. Plots of the observed diffusion coefficients of cAMP in 0.5% (w/v) agarose gel (●) and in 20% (w/w) Pluronic F127 gel (▲) at 37°C as a function of the reciprocal time. Each data point represents the mean and SD (n = 3). The lines represent a simple linear regression and the y-intercept of the regression lines were the corrected diffusion coefficients.
